# A new fasciocutaneous flap model identifies a critical role for endothelial Notch signaling in wound healing and flap survival

**DOI:** 10.1038/s41598-023-39722-1

**Published:** 2023-08-02

**Authors:** Khaled Dastagir, Jaba Gamrekelashvili, Nadjib Dastagir, Anne Limbourg, Dustin Kijas, Tamar Kapanadze, Peter M. Vogt, Florian P. Limbourg

**Affiliations:** 1 Vascular Medicine Research, Department of Nephrology and Hypertension, Hannover Medical School, Carl-Neuberg-Strasse 1, 30625 Hannover, Germany; 2 Department of Plastic, Aesthetic, Hand and Reconstructive Surgery, Hannover Medical School, Hannover, Germany; 3 The Jackson Laboratory, Bar Harbor, ME USA

**Keywords:** Angiogenesis, Inflammation, Inflammation

## Abstract

Flap surgery is a common treatment for severe wounds and a major determinant of surgical outcome. Flap survival and healing depends on adaptation of the local flap vasculature. Using a novel and defined model of fasciocutaneous flap surgery, we demonstrate that the Notch ligand Delta-like 1 (Dll1), expressed in vascular endothelial cells, regulates flap arteriogenesis, inflammation and flap survival. Utilizing the stereotyped anatomy of dorsal skin arteries, ligation of the major vascular pedicle induced strong collateral vessel development by end-to-end anastomosis in wildtype mice, which supported flap perfusion recovery over time. In mice with heterozygous deletion of Dll1, collateral vessel formation was strongly impaired, resulting in aberrant vascularization and subsequent necrosis of the tissue. Furthermore, Dll1 deficient mice showed severe inflammation in the flap dominated by monocytes and macrophages. This process is controlled by endothelial Dll1 in vivo, since the results were recapitulated in mice with endothelial-specific deletion of Dll1. Thus, our model provides a platform to study vascular adaptation to flap surgery and molecular and cellular regulators influencing flap healing and survival.

## Introduction

Flap surgery is a common reconstructive treatment for large wounds or defective organs. Flap surgery involves transfer of a donor tissue, such as a skin flap, supplied by a single major artery at its base into an injured recipient site. Survival and healing of the transplanted autologous tissue depends on maintenance and adaptation of the local flap vasculature to sustain flap perfusion^1^. The tissue region supplied by this principle artery is called angiosome^2^, which is a determinant of size and shape of the transplanted flap^3^.

The severance of blood supply from neighboring angiosomes during surgery induces ischemia and ischemic tissue damage^4,5^, which triggers inflammation, but also, as a compensatory response, angiogenesis and vascular collateralization from the principle artery, called arteriogenesis ^3,6^. Arteriogenesis occurs through the primary remodeling of small, preexisting arteries into large conductance vessels capable of maintaining or restoring perfusion^7^. It is regulated by intravascular signaling events originating in the endothelium and involves recruitment of monocytes and macrophages to ischemic vessels, which is essential for arteriogenesis^7,8^. However, the molecular regulation of arteriogenesis in fasciocutaneous arteries during flap transplantation surgery is poorly understood, in part because defined anatomical mouse models are lacking.

Canonical Notch signaling is an evolutionary conserved, cell–cell-contact dependent signaling pathway, which is activated by interaction of a membrane-bound Notch receptor with a Notch ligand expressed on an adjacent cell^9^. Notch is a key player in vasculo- and angiogenesis during development^9–11^, but also regulates arterial phenotype and arteriogenesis in peripheral arteries in the adult^12,13^. Furthermore, Notch also regulates myeloid cell fate during steady state and inflammation, particularly the anti-inflammatory differentiation of monocyte-derived macrophages during ischemia^8,14^.

The Notch ligand Delta-like 1 (Dll1) is selectively expressed by endothelial cells of large arteries and Dll1 haploinsufficiency leads to severely impaired arteriogenesis and ischemic tissue damage in a mouse model of hind limb ischemia^12^. In this setting, endothelial Dll1 regulates macrophage differentiation and maturation from invading monocytes, which promotes arteriogenesis and tissue repair and restrains inflammation after ischemia^8^.

Here, we studied the role of Notch signaling on flap vascularization and inflammation using a novel and anatomically defined fasciocutaneous flap model in mice with Dll1 loss-of-function. We demonstrate that Dll1 is required for collateral vessel formation and flap survival.

## Results

### A mouse skin flap model to study flap healing and arterial adaptation

**Figure 1:**
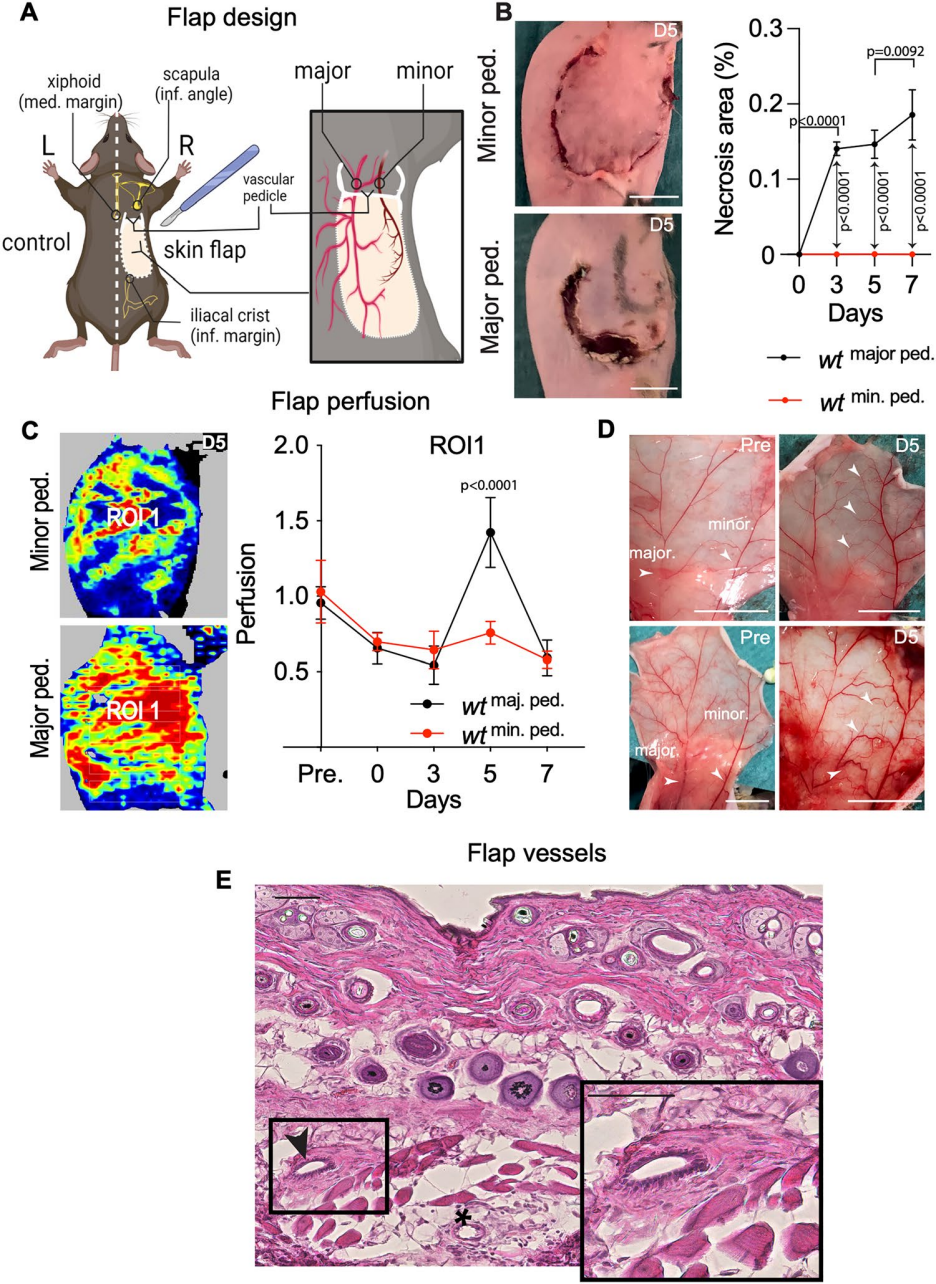
Mouse model for skin flap survival and arterial adaptation. (**A**) Illustration of flap design containing two blood vessels (major and minor pedicle). (**B**) Increased flap margin necrosis after ligation of the major pedicle on the fifth postoperative day (D5). Scale bar = 1 cm. Percent of flap necrosis area at D3, 5 and 7. (**C**) Flap perfusion measured by laser doppler following pedicle ligation, representative laser doppler images (LDI) of collateral circulation at D5. Percentage of flap perfusion compared to pre-operative control. (**D**) Pre-operative images (Pre) of the major and minor pedicles. Ligation of the major pedicle induces blood vessel collateralization at D5. Scale bar = 1 cm. (**E**) Representative H&E of collateral arteries. Arrow indicates vessel with enlarged *tunica media*. Scale bar = 50 μm. Statistical differences were assessed using a two-way ANOVA and Bonferroni post-test; n = 6/cohort; mean ± SEM;.

To study the contribution of angiogenesis and inflammation to surgical flap healing and survival we first developed a new surgical flap model, utilizing the stereotyped anatomy of dorsal skin arteries. In this, a dorsal skin flap containing two vascular pedicles (major and minor) is generated and re-sutured after ligation of one pedicle (Fig. 1A,B). Wound healing over time is quantified by wound necrosis area (Fig. 1B) while flap perfusion is monitored over time by Laser Doppler perfusion imaging (LDPI, Fig. 1C). Ligation of the minor pedicle did not result in development of significant (*p* = 0.0082) post-surgical necrosis, while flap perfusion was permanently, but significantly reduced (Fig. 1B,C). In contrast, ligation of the major vascular pedicle led to significant flap necrosis development at d3, followed by a slower expansion. At the same time, flap perfusion decreased until d3 in a similar manner as minor ligation but showed a significant increase over baseline values peaking at d5 (Fig. 1C), suggesting rapid and significant collateral vessel development in this variant and timepoint.

In order to assess the vascular status at d5, the flap was lifted and analyzed microscopically. After minor vessel ligation, perfusion of the minor pedicle occurred via fine end-to-end anastomoses, which connected minor and major pedicles (Fig. 1D, arrowheads). However, after ligation of the major vessel, reperfusion of the major vessel occurred by formation of large and tortuous collateral blood vessels from the intact minor pedicle in an end-to-end fashion (Fig. 1D, arrows), which, by histologic examination, displayed a thicker vessel wall compared to native arteries of the flap (Fig. 1E). These data suggest that major pedicle ligation provides a robust and reproducible stimulus for collateral artery formation in a skin flap model, which allows for studying wound healing and flap survival. We therefore chose this variant for further investigations of blood vessel regeneration and inflammation in flaps.

### Notch ligand Dll1 regulates flap collateral vessel development and wound healing

**Figure 2:**
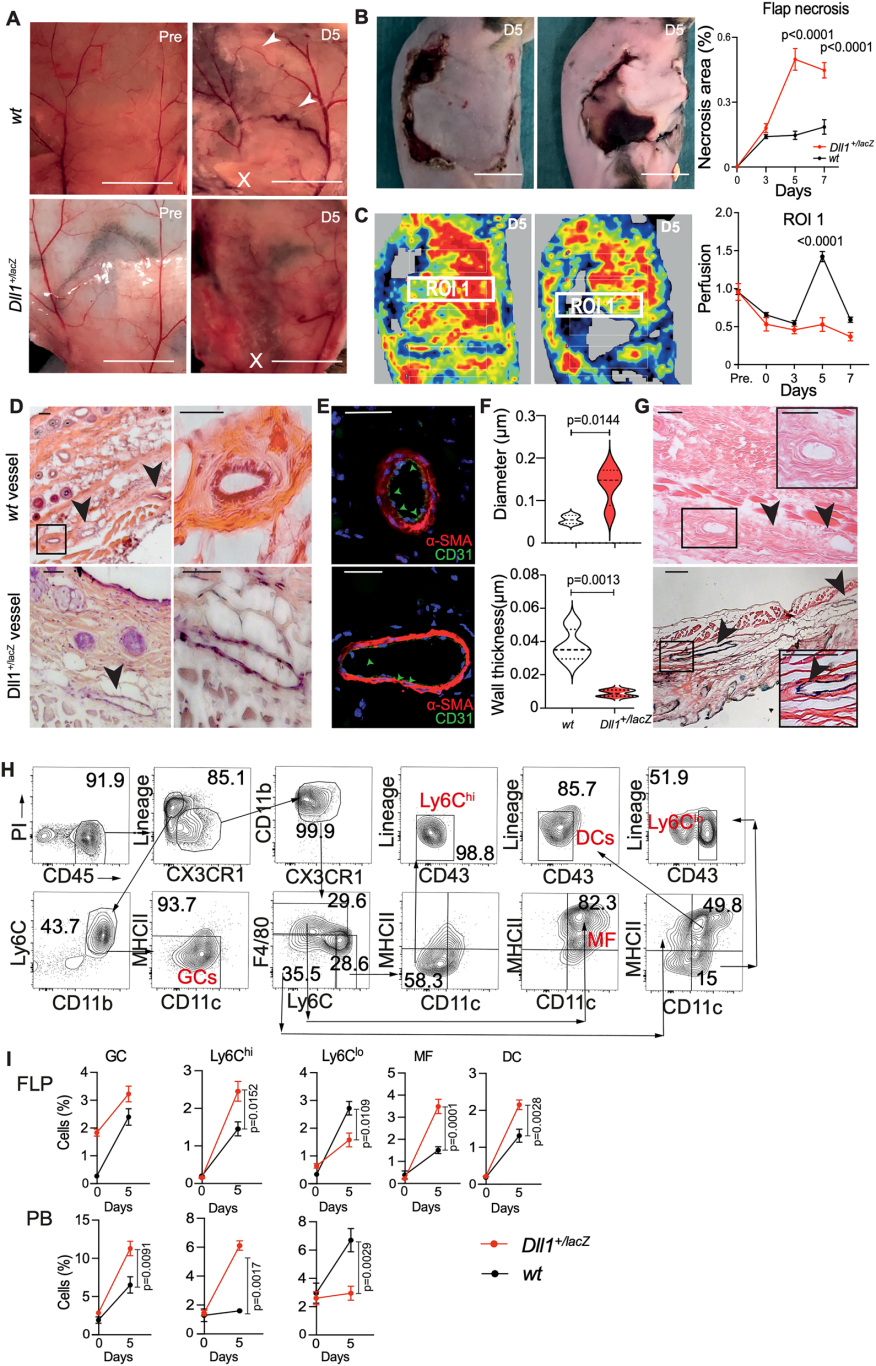
Notch ligand Dll1 regulates flap collateral vessel development, inflammation and wound healing. (**A**) Preoperative (Pre) images demonstrate the two intact (minor and major) pedicles of the flaps in WT and *Dll1*^+*/lacZ*^ mice*.* Representative images at D5 after ligation of the major pedicles (X) show insufficient collateral vessel formation in *Dll1*^+*/lacZ*^ mice. Arrows mark collateral vessels. (**B**) Increased flap necrosis in *Dll1*^+*/lacZ*^ mice compared to wild-type mice after ligation of the major pedicle at D5. A&B scale bar = 1 cm. Percentage of flap necrosis area at D5. (**C**) Laser doppler images (LDI) show collateral circulation at D5. (**D**) Representative H&E staining of collateral arteries in WT and *Dll1*^+*/lacZ*^ mice*.* Insufficient vessel formation is observed in *Dll1*^+*/lacZ*^ mice at D5. Arrows mark arteries. (**E**) Immunofluorescent staining of SMA- (red) and CD31 (green) in flap sections at D5 after ligation of the major pedicle. (**D**, **E**) Scale bar = 50 μm. (**F**) Quantification of collateral vessel artery inner circumference and wall area using H&E staining at D5. (**G**) LacZ staining of collateral arteries in WT and *Dll1*^+*/lacZ*^ shows insufficient vessel formation in *Dll1*^+*/lacZ*^ mice at D5. Arrows mark arteries. Statistical differences assessed using two-way ANOVA and Bonferroni post-test and unpaired student t-test as appropriate. n = 6/cohort, mean ± SEM. (**H**) Flow cytometry gating strategy for various myeloid cell populations. (**I**) Flow cytometric analysis of ischemic flap from *Dll1*^+*/lacZ*^* vs WT* mice baseline (0) and at D5. Percentage of different myeloid subpopulations in flap (Flp) and peripheral blood (PB) of WT and *Dll1*^+*/lacZ*^ mice are shown (n = 9 biological replicates/cohort).

We next analyzed the impact of Notch signaling on arteriogenesis and flap necrosis (Fig. 2A–C). To this end, we employed mice with general-heterozygous deletion of *Dll1*, a ligand for Notch receptors expressed by endothelial cells^12^, in which one allele is replaced by insertion of lacZ (*Dll1*^+*/lacZ*^). At baseline, the vascular architecture of the flap was comparable between wt and *Dll1* mutant mice, as *Dll1*^+*/lacZ*^ show no general baseline phenotype. However, five days after major pedicle ligation, Dll1 mutant mice showed a striking deficiency of collateral vessel formation from the intact minor pedicle and hypoperfusion of the major pedicle and its territories (Fig. 2A). Consequently, flap wound healing was strongly impaired, leading to significantly increased flap necrosis after d3 (Fig. 2B). Furthermore, flap perfusion by LDPI, which increased as expected around d5 in wt mice, remained significantly depressed in *Dll1* mutant mice until d7. We next studied collateral vessel architecture in flap sections. Compared to wt mice, the collateral arteries in subepidermal layers of the flap appeared enlarged, but with markedly thinner vascular wall comprised of vascular smooth muscle cells, which was corroborated by quantification of collateral vessel diameter and wall thickness (Fig. 2D–F). This demonstrates impaired and aberrant vessel adaptation and remodeling in response to skin ischemia in *Dll1* mutant mice. To address, whether the atypical collateral vessels in mutant mice were descendants of arteries, we employed β-galactosidase staining, since in *Dll1*^+*/lacZ*^ mice, β-galactosidase, the gene product of lacZ, is specifically expressed in Dll1-expressing cells^12^. In mutant mice, there was strong staining in atypical vessels in the subepidermal layers, and sparse staining in epidermal hair follicles, while no signal was detected in wt mice, demonstrating specific staining of the reporter allele and arterial origin of aberrant vessels in *Dll1*^+*/lacZ*^ mice (Fig. 2G, Supplemental Fig. 1).

### Dll1 loss of function promotes a myeloid inflammatory response

Ischemia causes a myeloid inflammatory response intended to repair damaged tissues and promote angiogenesis, but can become destructive when unregulated^8^.To characterize the inflammatory response, flow cytometry was performed around the time of perfusion boost (d5) with a dedicated myeloid panel in wildtype and Dll1^+*/LacZ*^ mice^14^. This gating strategy defined five different populations based on surface marker profiles, defining Ly6C^hi^ and Ly6C^lo^ monocytes, macrophages (MF), dendritic cells (DC) and granulocytes (GC) (Fig. 2H,). While there was no difference between mutant and wt mice in skin-resident cell populations at baseline, Dll1 haploinsufficient mice showed a markedly altered response to surgery in the skin flap, but also systemically. The frequencies of Ly6C^hi^ monocytes, which give rise to macrophages^8^, Ly6C^lo^ monocytes, dendritic cells, and macrophages were markedly and significantly increased in the flap of Dll1^+*/LacZ*^ mice compared to wildtype controls (Fig. 2I), demonstrating a severe pro-inflammatory response. In addition, mutant mice showed a systemic response reflected by significant elevation of circulating Ly6C^hi^ monocytes and GC at d5 (Fig. 2I).

**Figure 3:**
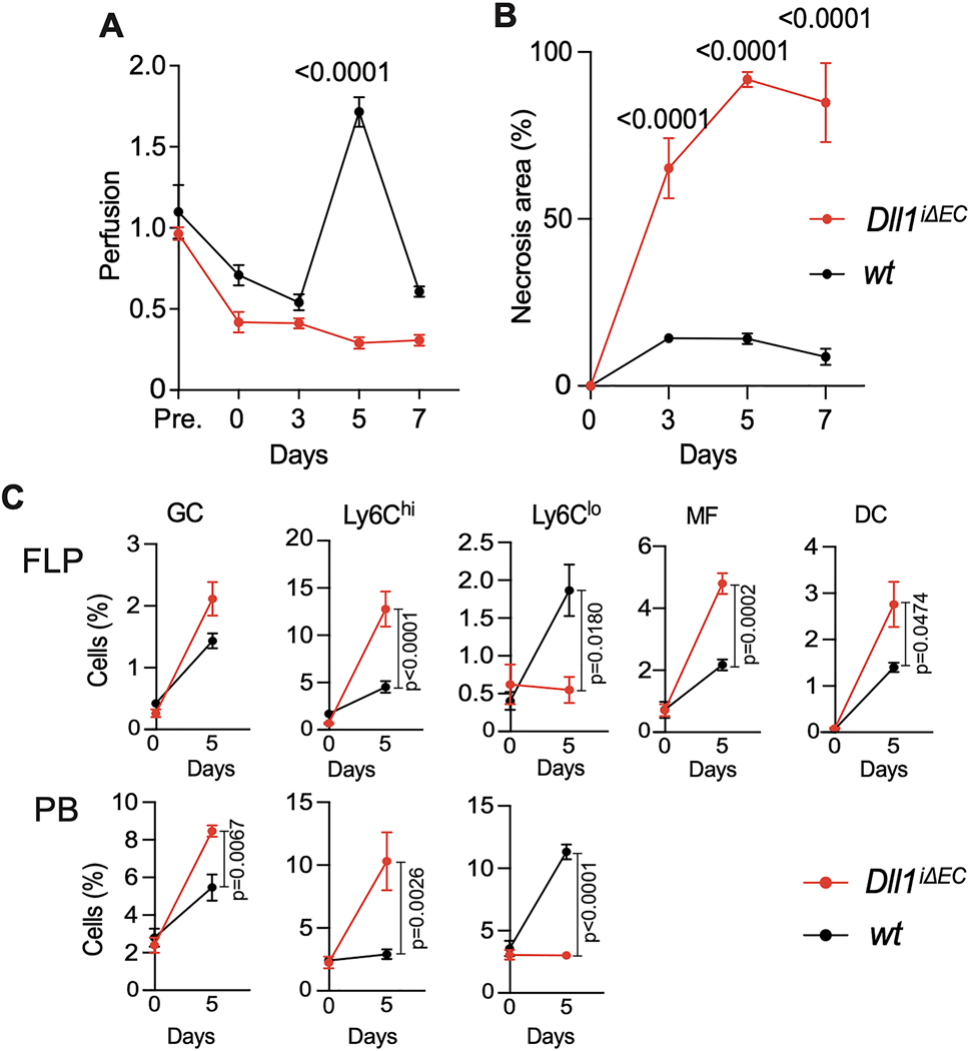
Role of endothelial Dll1 in post-ischemia wound healing and inflammation. (**A**) Flap perfusion by laser Doppler perfusion measurement in *WT* mice vs mice with endothelial-specific deletion of *Dll1* (*Dll1*^*iΔEC*^*)* 5 days after ligation of each pedicle. n = 6/cohort, mean ± SEM. (**B**) Percent necrosis area at D5, n = 6/cohort, mean ± SEM. (**C**) Representative flow cytometric analysis of ischemic flap (FLP) of *Dll1*^*iΔΕC*^* vs WT* mice baseline (d0) and at d5. Percentage of different myeloid subpopulations in FLP and PB of WT and *Dll1*^*iΔEC*^ mice are shown (n = 9 biological replicates/cohort). Statistical differences assessed using a two-way ANOVA and Bonferroni post-test.

Since the Notch ligand Dll1 was expressed in vascular endothelial cells, but also extravascular cells in skin (Fig. 2G), we next tested the hypothesis that endothelial Dll1 regulates flap perfusion, inflammation and survival. To this end we used endothelial-specific and inducible Dll1 mutant mice (*Dll1*^*iΔEC*^), which carry conditional Dll1 alleles and the Cdh5(PAC)-creERT2 transgene for tamoxifen-inducible cre activation^8,14^. In controlled experiments, endothelial Dll1 mutant mice showed a strongly impaired perfusion response to flap surgery, resulting in severe flap necrosis (Fig. 3A,B). Furthermore, recruitment of Ly6C^hi^ monocytes to the ischemic region and development of macrophages in the flap was strongly increased in Dll1 mutant mice, while recruitment of Ly6C^lo^ monocytes was strongly impaired (Fig. 3C). Infiltration of inflammatory cells into the flap region was accompanied with increased numbers of blood circulating Ly6C^hi^ monocytes and GC.

## Discussion

Using a novel and defined murine model of large flap surgery, we demonstrate that the remodeling of preexisting collateral vessel networks, is critical for flap survival. Furthermore, we found evidence that arteriogenesis in flaps is dependent on Notch signaling triggered by endothelial-specific Notch ligand Dll1. The loss of Dll1 function resulted in impaired arteriogenesis and perfusion recovery accompanied by severe inflammation due to enhanced recruitment of Ly6C^hi^ monocytes.

Experimental flap models have advanced application and understanding of surgical techniques, yet the current models need further refinement to characterize molecular mechanisms regulating vascularization success. An advantage of flap models in large animals, such as pigs or sheep, is that they allow the performance of multiple flaps per animal^15^. However, flap models in rodents are beneficial because these animals are more disease resistant, cost-effective, easier to acquire and have genetic tractability. Notably, mice offer an almost inexhaustible potential of genetic modification. Many flap model variations have been constructed in small rodents such as abdominal cutaneous flaps, McFarlane flap (cranially based and randomly perfused dorsal skin flap) and dorsal skinfold chamber. These models have been used to define flap anatomy, perfusion, physiology and to improve surgical techniques^15^. However, these studies did not develop a model of inducible, controlled ischemia where postoperative development of collateral vessels can be examined.

Therefore, we established a new mouse flap model which allows investigation of vascular regeneration and inflammation on the molecular level. Previous flap models are difficult to replicate and require advanced surgical skills. Our newly established mouse flap model facilitates investigation of vascular regeneration and inflammation due to defined blood vessel supply and simple preparation. After ligation of the major pedicle we observed hyperperfusion of the flap and collateral vessel formation sprouting from the minor pedicle on the fifth postoperative day. Due to the new collateral vessels the flap was sufficiently reperfused, indicating the importance of arteriogenesis (Fig. 1D, 19,22).

After establishing and validating the use of our flap model, we next examined the molecular regulation of vessel adaptation in relation to Notch signaling. Using haplodeficient and endothelial-specific deletion of Dll1 we demonstrate the ligand’s key role in vascular collateralization, regeneration and tissue wound healing. Dll1^+*/LacZ*^ mice showed impaired collateralization resulting in impaired reperfusion of the flap. As a consequence of reduced vascularization, a larger necrosis area was observed in mutant flaps compared to wildtype controls. This suggests decreased Dll1 function affects vascular formation and blood supply to the flap resulting in poor healing.

One of the angiocrine functions of blood vessels is their involvement in tissue maintenance and regeneratio^16^. Local endothelial cells express instructive cues after tissue injury and orchestrate the response of tissue resident progenitor cells. The fate of these progenitor cells is influenced by Notch signaling. In our study we investigated the role of Dll1 in arteriogenesis and regeneration by using a controlled ischemia model. It is already known that Dll1 expression is upregulated in arterial endothelial cells due to ischemia^12^. Furthermore, Dll1 activates both Notch1 and Notch2, however it induces higher Notch2 activity than Notch1^17^. Upregulation of Notch2 has been associated with differentiation of monocytes from an Ly6C^hi^ characterization to Ly6C^lo^^14^. The observed increase of Ly6C^hi^ monocytes in Dll1^+/lacZ^ mice corresponds with this depletion of Notch Dll1 signaling. Ly6C^lo^ monocytes are associated with vascular formation and improved regeneration^8^. This decreased monocyte subpopulation resulted in poor flap healing outcomes.

Comparative analysis of the vascular phenotype of collateral vessels in Dll1 deficiency revealed rudimentary and incomplete formation of blood vessels in Dll1^+*/lacZ*^ reporter mice, showing a thin wall architecture. In particular, the formation of the collateral vessels’ tunica media seemed to be disturbed. These results demonstrate a critical role for Dll1 in vascular arterial remodeling in flaps. The fact that vascular-endothelial targeting of Dll1 phenocopied the general heterozygous phenotype suggest that endothelial Dll1 is critical for this process, which also makes extra-endothelial contributions from other cell types unlikely, e. g. myeloid cells^18^. However, although we did not find evidence of lymphatic expression of Dll1 in the skin, we cannot fully excluded a lymphatic-endothelial contribution. Collectively, these results demonstrate the Notch signaling plays a direct role in arteriogenesis, regulated by endothelial Dll1 expression, which may act on vascular or myeloid cells, the latter being an important immune cell involved in vascular repair.

The inferior collateralization response in Dll1-mutants was accompanied by enhanced mobilization and expansion of inflammatory cells, including monocytes, and granulocytes in the flap and peripheral blood compared to controls (Fig. 3). The increased inflammatory cell populations in the ischemic tissue, accompanied with under expression of Dll1, is recapitulated in a hindlimb ischemia model^8^. One reason for the overwhelming inflammatory reaction in Dll1-deficient mice may be the lack of interaction between endothelial cells and monocytes. Dll1 expression in endothelial cells has been shown to coordinate monocyte fate^8,14^. Another reason for the increased inflammatory response might be the large flap necrosis. Our data suggests a large influx of immune cells in the flap is likely to inhibit the repair process. Since there is more necrosis some of the useful functions of the immune cells, such as phagocytic activity, may be reduced. However, further studies are needed to understand the exact Notch-regulated mechanisms of this inflammatory response and immune cell interactions that affect arteriogenesis.

Use of this novel and defined mouse flap model may set the stage to specifically address the processes of arteriogenesis, angiogenesis, inflammation and wound healing. It may also promote studies on the molecular and cellular events involved in flap failure and flap survival. Furthermore, by demonstrating the critical involvement of Notch signaling mediated by the Dll1 ligand, our study may point to new prognostic factors or therapeutic options to promote flap tissue recovery from vascular injury and restoring blood supply to ischemic tissues.

## Material and methods

### Mice

Animal experiments were approved by the ethics committee of the state animal welfare board of Niedersachsen (LAVES, TVA18/2969). All experiments were performed in accordance with relevant guidelines and regulations and executed in compliance with state animal protection laws (TierSchG) and the ARRIVE guidelines (https://arriveguidelines.org). The Dll1^+*/LacZ*^ mice (129-*Dll1*^*tm1Gos*^/J) and *Cdh5-cre-ERT2;Dll1*^*f/f*^ (B6;129-Tg(Cdh5(PAC)-cre/ERT2)1Rha *Dll1*^*tm1Mjo*^) mice have been described^12,19^ and were housed under specific pathogen-free conditions at Hannover Medical School. 10–12 weeks-old male mice and age- and sex-matched littermate controls were used in experiments. Tamoxifen-regulated cre-recombinase activation and deletion of *Dll1* in *Cdh5-creERT2;Dll1*^*f/f*^ (Dll1^iΔEC^ mice) was induced as described^20^ two weeks before surgical intervention.

### Flap model

Mice were anaesthetized with a mixture of Ketamine 80 mg/kg (Pfizer, Berlin, Germaniy), Xylazine 2.5 mg/kg (Bayer healthcare, Leverkusen, Germany), and Midazolam 2.5 mg/kg (Hexal, Holzkirchen, Germany). Briefly, a fasciocutaneous flap with one major and one minor pedicle was isolated from the right side of the mouse (Fig. 1a). The major or minor pedicle (artery) was surgically ligated using Marlin 6.0 Catgut GmbH, Markneukirchen, Germany) distal to its origin from axillary artery. The flap was then placed back on the wound and the skin was sutured using Mariderm 6.0 (Catgut GmbH, Markneukirchen, Germany) in a continuous and intracutaneous technique. In compliance with German Animal Welfare guidelines, Novaminsulfone (Novalgin 1.6 ml/liter) supplemented water was provided for the animals via drinking water for 24 h prior to the surgeries and for the remaining postoperative experimental time points.

### Flap perfusion

Flap perfusion, as region of interest (ROI), and the perfusion of the contralateral side, as negative control, were measured immediately after surgery and 1, 2 and 5 days postoperatively using Perimed LDPI PIM II Laser Scanner (Perimed, Sweden). Perfusion of flap was calculated as ratio of flap vs. contralateral side.

### Necrosis area

The flap was photographed preoperatively, immediately after surgery and, 1, 2 and 5 days postoperatively under standard conditions. Total flap- and necrosis areas were measured using Fiji ImageJ software (1.53d 19 August 2020)^21^. Percentage of necrosis area was calculated.

### Collateral vessels

The growth of collateral blood vessels was assessed and photographed intraoperatively at d0 and 5 days postoperatively.

### Tissue histology and immunohistochemistry

Immunohistochemistry, H&E, LacZ staining and immunofluorescence staining in mice were performed with modifications from previous descriptions^8,12,14^. Flap and contralateral side were excised, incubated in 15% (6 h) and 30% (18 h) sucrose and embedded in Tissue-tek OCT compound (Sakura, California, USA). Slides were co-stained with H&E and analyzed with Olympus IX71 microscope. The arterial wall and Lumen area was calculated using ImageJ software (National Institutes of Health, Bethesda, USA). For immunofluorescence (IF) and confocal laser scanning microscopy (CLSM) tissue sections were stained using anti-SMA, anti-CD31, anti-LYVE1 and appropriate fluorescence-conjugated secondary antibodies. Nuclei were counterstained using DAPI and slides were mounted in fluorescence mounting medium (DAKO). Images were acquired using Leica TCS SP2 AOBS (Leica Microsystems, Germany) confocal microscope or Zeiss Observer Z1 fluorescence microscope (Zeiss, Germany) respectively.

### Flow cytometry

To prepare single-cell suspensions for flow cytometry, the flap and fasciocutaneous tissue from the contralateral side were excised and digested in DMEM containing 2 mg/ml collagenase II (type 2, derived from Clostridium histolyticum, Catalog no#LS004176, Worthington Biochemical Corp.) at 37 °C for 20 min using Gentle MACS dissociator (Miltenyi). Total viable cell number was determined in digests with Trypan blue in a Neubauer chamber. Non-specific binding of antibodies to Fc-receptors was blocked using anti-mouse CD16/CD32 (TruStain fcX from BioLegend) in single-cell suspensions from peripheral blood (PB), skin of the flap and control side. After subsequent washing step, cells were labeled with primary and secondary antibodies and were used for flow cytometry analysis (LSR-II, BD Biosciences). For apoptosis assay, single-cell suspensions were stained with primary and secondary antibodies, washed, re-suspended in AnnexinV binding buffer (Biolegend) and transferred into tubes. Cells were stained with AnnexinV (AnnV) and propidium iodide (PI) at room temperature for 20 min and were immediately analyzed by flow cytometry. Antibodies and fluorochromes used for flow cytometry are described in (Table 1). Flow cytometry data were analyzed using FlowJo software FlowJo LLC). Initially cells were identified based on FSC and SSC characteristics (Table 2). After exclusion of doublets (on the basis of SSC-W, SSC-A), relative frequency of each subpopulation from live cell gate, or absolute number of each subset (calculated from live cell gate and normalized per mg Skin of flap or control side, or per ml PB) were determined and are shown in the graphs as mean ± SEM, unless otherwise stated.

**Table 1:** List of antibodies used for flow cytometry.

Antibody	Clone	Label	Dilution	Company	Cat. #
CD32/16	93	Unlabeled	1:200	Biolegend	101,319
CD45	30-F11	AF700	1:400	Biolegend	103,128
F4/80	BM8	APC	1:100	Biolegend	123,116
CX3CR1	SA011F11	PE	1:200	Biolegend	149,005
CD19	6D5	Bio	1:400	Biolegend	115,504
B220	RA3-6B2	Bio	1:400	Biolegend	103,203
CD3	17A2	Bio	1:200	Biolegend	100,243
Ter119	Ter119	Bio	1:400	Biolegend	116,203
NK1.1	PK136	Bio	1:200	Biolegend	108,704
Ly6G	1A8	Bio	1:400	Biolegend	127,603
CD11b	M1/70	Pacific Blue	1:400	Biolegend	101,224
Ly6C	HK1.4	PE-Cy7	1:1400	Biolegend	128,018
I-A/I-E	M5/114.15.2	BV510	1:400	Biolegend	107,635
CD11c	N418	BV605	1:400	Biolegend	117,334
CD43	S7	PerCP-Cy5.5	1:400	BD Pharmingen	562,865
LYVE1	ALY7	Biotin	1:200	ThermoFisher	Ab_1724157
Streptavidin		PE-Dazzle 594	1:400	Biolegend	405,247

**Table 2:** Overview of gating strategies.

Population	Phenotype
Ly6C^hi^	CD45^+^Lin^-^CD11b^+^CX_3_CR1^+^Ly6C^hi^F4/80^lo/-^CD11c^-^MHC-II^lo/-^CD43^-^
Ly6C^lo^	CD45^+^Lin^-^ CD11b^+^CX_3_CR1^+^Ly6C^lo/-^F4/80^lo/-^CD11c^lo^MHC-II^lo/-^CD43^+^
MF	CD45^+^Lin^-^ CD11b^+^CX_3_CR1^+^Ly6C^lo/-^F4/80^hi^CD115^+^
DC	CD45^+^Lin^-^CD11b^+^CX_3_CR1^+^Ly6C^lo/-^F4/80^lo/-^CD11c^+^MHC-II^+^CD43^-^
GC	CD45^+^Lin^+^CD11b^+^CX_3_CR1^-^Ly6C^lo^F4/80^-^CD11c^-^MHC-II^-^

### Statistical analysis

Results are expressed as mean ± SEM. n numbers indicate biological replicates of N > 3 experiments performed at least three times unless otherwise indicated. For comparison of multiple experimental groups two-way ANOVA with Bonferroni’s multiple comparison post-test was performed.

## Supplementary Information

Supplementary Information.

## Data Availability

The data is available from the corresponding author upon reasonable request.
