# Supplementary material for: A new fasciocutaneous flap model identifies a critical role for endothelial Notch signaling in wound healing and flap survival
Source: Sci Rep. 2023 Aug 2;13:12542. doi: 10.1038/s41598-023-39722-1 (PMC10397185; doi:10.1038/s41598-023-39722-1)
Supplement: Supplementary file 1 — Supplementary Information. [file 41598_2023_39722_MOESM1_ESM.pdf]

A new fasciocutaneous flap model identifies a critical role for endothelial Notch signaling in wound healing and flap survival

Khaled Dastagir[1,2], Jaba Gamrekelashvili[1], Nadjib Dastagir[1,3], Anne Limbourg[1,2], Dustin Kijas[1], Tamar Kapanadze[1], Peter M. Vogt[2], Florian P. Limbourg[1]

Supplemental Data:

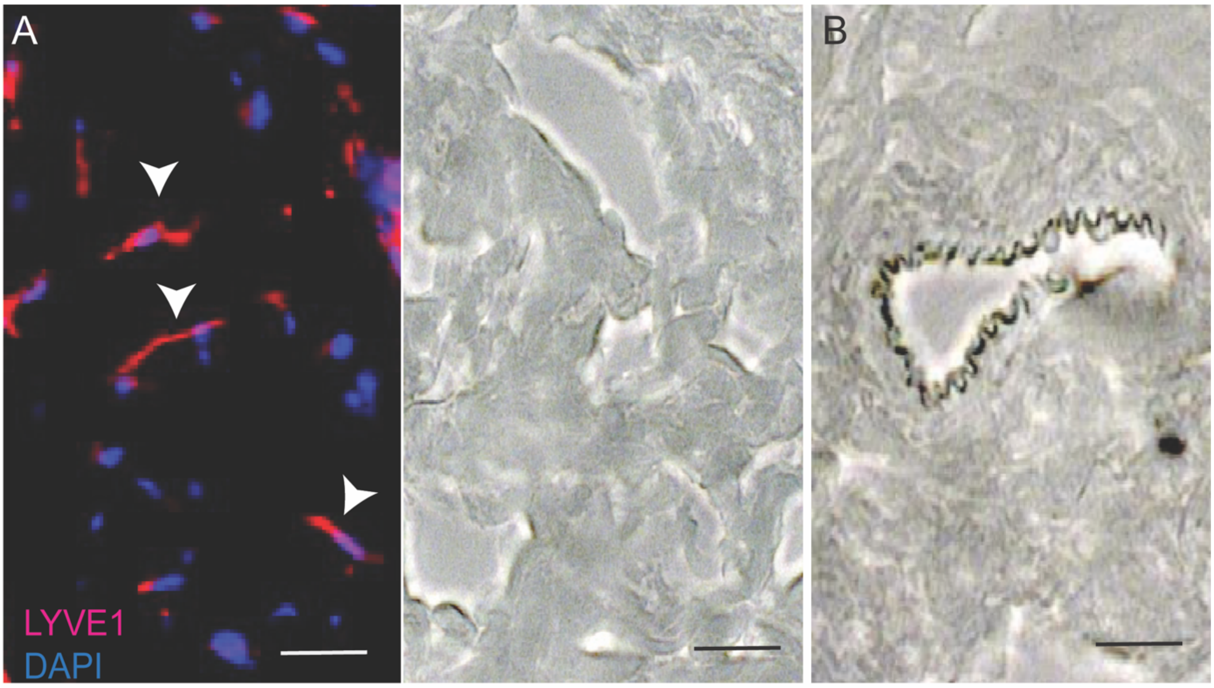

Supplemental Fig 1. Dll1 expression in endothelial cells in the skin in *Dll1*$^{LacZ/+}$ mice. (A) Immunofluorescent and LacZ staining in the same representative flap section at D5 after ligation of the major pedicle. Left: LYVE1- (red) and DAPI (blue). Arrows indicate lymphatic cells. Right: LacZ staining. (B) LacZ staining (black) in unrelated flap section including a collateral artery. Scale bar=20µm.
